# Is Pain a Symptom or a Disease? How Does the New Evidence Help to Better Understand this Unsolved Question?

**DOI:** 10.2174/1570159X2201230921165106

**Published:** 2023-09-21

**Authors:** Massimo Allegri

**Affiliations:** 1 Centre lémanique de neuromodulation et thérapie de la douleur – Ensemble Hospitalier de la Côte – Morges – Switzerland

Pain is a complex phenomenon with a complex neurophysiology. Recently, the definition of pain has been revised, and a new classification has been proposed to enhance its definition, aiding in improved diagnosis and understanding of this phenomenon [1]. In fact, pain could be a simple symptom of an acute or chronic disease, or it could be a disease by itself with an autonomous pathophysiology as its basis. For example, the new entity “nociplastic pain” confers to several syndromes. One of them is fibromyalgia, a new classification that could help to understand how the alterations in pain pathways could lead to a distinct disease, with pain itself being a prominent symptom. On the other hand, it is important to differentiate this new entity from what we have defined for several years as “spinal sensitization” [2]. Another important issue is represented by persistent pain after surgery [3], a pain that lingers even after 3-6 months post surgery that can affect up to 20% of people after surgery, where only a better understanding of the pathophysiology of how acute pain becomes chronic can lead the physicians to treat this syndrome efficiently.

A new important role of the immune system in transitioning from a symptom to a chronic disease has been recently demonstrated in chronic persistent pain after surgery [4] and almost in chronic syndromes such as low back pain and temporomandibular pain [5]. Interestingly, these two articles have underlined how chronic pain is not an aberrant response to a painful stimulus, as thought until now, but it is exactly the opposite: the disease of chronic pain and the persistence of pain over time is the “non-response” of the immune system. This new discovery highlights the strict interaction between neurons and immune systems and how the incorrect response of the immune system can determine modification of the nervous system, leading to a disease called chronic pain. These new insights open. Also, in the case of neuropathic pain, there exists a strict relationship with the immune system [6], especially, in this case, with T cells. All these discoveries are paving new scenarios both to prevent the development and to treat chronic pain, focalizing new possible therapeutic targets. In this context, regenerative medicine could be used not only for its “regenerative” properties but for its long-lasting “immunomodulatory and antiinflammatory” properties [7].

Unfortunately, despite all these new discoveries and all new therapies proposed, there is still a big unmet need to understand better pain pathophysiology and how we can treat it appropriately. Hence, in this thematic issue, we propose, through the contribution of some of the most important experts in the “Pain Field,” a series of articles will try to deepen some aspects of the new evidence of pain pathophysiology and therapy with a bench-to-bedside approachIn fact, only by linking even stronger the “bench” studies to clinical practice we will have the opportunity to understand better and treat our chronic pain patients. By delving into the emerging evidence on how we can modulate the pathophysiology of the peripheral and central nervous system, we can explore potential new treatments for both acute and chronic pain.

This thematic issue will be addressed to deepen new insights into pain pathophysiology and how they can define a new possible target for therapy not only for pain as a symptom but also for pain as a disease with a special focus on nociplastic pain, a “new” taxonomic entity that needs more careful attention in treating chronic pain. Finally, this special issue will focalize on how new integrators, drugs and interventional techniques can modulate pain, always evaluating not only the clinical effect (“the pain relief”) but also the “pathophysiological effect” in order to understand how the new discoveries of today will be the basis of the research in the future.
